# New Uses for Veratrum Viride

**Published:** 1879-06-20

**Authors:** M. E. Bishop

**Affiliations:** South Haven, Mich.


### *NEW USES FOR VERATRUM VIRIDE—VERATRUM VIRIDE IN SCARLATINA.*

BY M. E. BISHOP, M.D., SOUTH HAVEN, MICH.

Having for some years made a special study of veratrum viride in zymotic diseases, I have been requested to give the result of my observations to the profession at large. I will, therefore, begin with scarlatina, which has hitherto proved particularly unmanageable. I consider veratrum viride more like a specific than all other remedies, since it invariably shortens the disease by one-half, and so modifies the sequelæ that they are easily managed. Since I began its use I have not lost a single patient—when I had the case from the beginning.

When called to a case, I begin with a medium dose; for a child five years of age, one drop of Tilden's fluid extract every two hours, then more or less, according to the severity of the case. The more serious the invasion, the more veratrum must be given, watching the case carefully until the pulse is brought down to a normal standard; which will soon follow, notwithstanding the unnatural heat induced by the presence of zymotic poison in the system. Veratrum not only neutralizes the poison, but reduces the action of the heart. I have often been surprised and gratified, on my second visit to the little fever-tossed sufferers, to find the skin moist and cool, and the pulse, which was one hundred and thirty at the first visit, now below one hundred. As a rule, it is never necessary or desirable to bring the pulse below the average of health, taking into consideration the age and temperament of the child; a deviation from the usual average would be apt to cause unpleasant symptoms, although veratrum may be given until it produces vomiting, without causing any serious depression.

The expression "more or less, according to the case," needs some explanation. I have attended cases of children, one and two years old, who would bear two drop doses several hours without any apparent effect, and after three or four days one-half drop would produce disagreeable symptoms. In those cases it was not from a continued or cumulative effect of the remedy, for I had stopped its use for twenty-four hours. So, I repeat that the dose must be proportioned to the amount of the zymotic poison present, and, when that poison is neutralized, a very small dose is sufficient to continue the effect. If there should be a return of the fever, after having discontinued the veratrum, renew the prescription, and give as at first. I have found, instead of four days to the height of the disease and four to the decline, it will be only four or five days in duration, with returning secretion and return of appetite.

Sometimes my little patients complain to me that they are not given enough to eat, twenty-four hours after I begin to give the medicine; and the friends would express fears that the eruption had "struck in," as they termed it, because it went off so soon.

Veratrum seems to exert a special influence upon the anginose form of the disease, reducing the swelling in a few hours. In such cases the remedy must be pushed till it causes considerable ptyalism. Sometimes this will be annoying, but can be easily arrested by giving a little tannic acid in solution. Tannic acid will arrest the vomiting from overdoses of veratrum the most readily of anything I have ever used—in fact, I have used nothing else for that purpose for the last ten years. More than half the vomiting caused by veratrum is by reason of its special effect on the throat, which might be compared to tickling the fauces, which tannic acid speedily relieves. It must be remembered that veratrum is not a restorative, and if given in an advanced case of scarlatina will not restore the blood to its normal condition, or act upon the kidneys. When I first began to use it, I thought, because none of the cases where it was used early developed the peculiar fetor of the disease, that it was an antiseptic. But since then I have come to the conclusion that it immediately destroys or neutralizes the zymotic poison. The sequelæ of scarlatina that have been treated with veratrum are almost always absent, or if the case has been unusually severe and some present themselves, they prove to be light and easily manageable.

It is hardly to be expected that a physician will always have every case of scarlatina before the child's parents have attempted to treat it themselves, and too often by an active cathartic, which will cause much debility, by causing the eruption to expend its force upon the mucous membrane of the alimentary canal. I am continually surprised to see how little debility follows the use of veratrum, which I account for by the fact that it neutralizes the poison before it produces any lesions of the nerve centres. When the fever runs high with active delirium, it is well to combine gelseminum with veratrum; if there is stupor, use belladonna; if there is vomiting, first give a stimulating emetic. If the child's throat is sore, with difficulty of swallowing, mix the veratrum with syr. acaciæ; for very young children it is best always to thus prepare it.

I cannot close this article without speaking of two remedies that I have used in the secondary stages or sequelæ of scarlatina: mur. tinc. ferri, and sulphite soda—which, if given according to the indications, never fail to speedily restore the blood to its normal condition. After using veratrum for five years, in numerous cases and in constitutions of the second and third class (whoever has read Byrd Powel on Temperaments will understand what is meant by second and third class), I can truly say I have found no remedy so near like a specific for scarlatina as veratrum viride. I say to my professional brethren, for hnmanity's sake, give it a fair trial.—*N. Y. Med. Journal.*

